# Presentation of Sjogren Syndrome as Nodular Pulmonary Amyloidosis

**DOI:** 10.7759/cureus.30103

**Published:** 2022-10-09

**Authors:** Harjinder Singh, Parth M Patel, Sruthi Ramanan, Hazem Zebda, Devin Malik

**Affiliations:** 1 Internal Medicine, Henry Ford Health System, Jackson, USA; 2 Rheumatology, Henry Ford Health System, Detroit, USA; 3 Hematology/Oncology, Henry Ford Health System, Jackson, USA

**Keywords:** localized amyloid, pulmonary nodular amyloidosis, amyloidosis, pulmonary disease, sjogren's disease

## Abstract

Sjogren syndrome is an autoimmune disorder that leads to dryness in the eyes and mouth. Nodular pulmonary amyloidosis is a localized amyloid deposition pathology commonly seen with monoclonal lymphoproliferative disorders. We present a patient who came in with dyspnea and was found to have nodular pulmonary amyloidosis on biopsy. Commonly associated lymphoproliferative pathologies were ruled out and on further workup, the patient was found to have Sjogren syndrome. This case demonstrates pulmonary nodular amyloidosis as a rare presentation of Sjogren syndrome in the setting of relatively well-controlled symptoms. Detection of pulmonary nodular amyloidosis should prompt evaluation of associated conditions such as malignancy and autoimmune disorders to guide further management.

## Introduction

Amyloidosis is a heterogeneous group of disorders associated with the deposition of proteins in an abnormal fibrillar form in tissues [1]. Light chain (AL) amyloidosis and reactive (AA) amyloidosis are the two most common types of amyloidosis. In AL amyloidosis, clonal plasma cells produce light chains (λ or κ) that are amyloidogenic [2]. AA amyloidosis occurs due to increased secretion of serum amyloid A (SAA) protein, a protein produced in inflammatory pathologies [3]. Along with the sustained elevation of SAA, genetic polymorphisms in SAA and the type of autoinflammatory syndrome facilitate conversion to SAA to beta-pleated sheet and deposition in organs [4]. Pulmonary involvement is usually insignificant in systemic amyloidosis, and localized pulmonary amyloidosis is infrequently reported [5]. Isolated pulmonary amyloidosis in Sjogren syndrome is also an uncommon phenomenon. Here we present a patient who was found to have pulmonary nodular amyloidosis with Sjogren syndrome. 

## Case presentation

**Figure 1:**
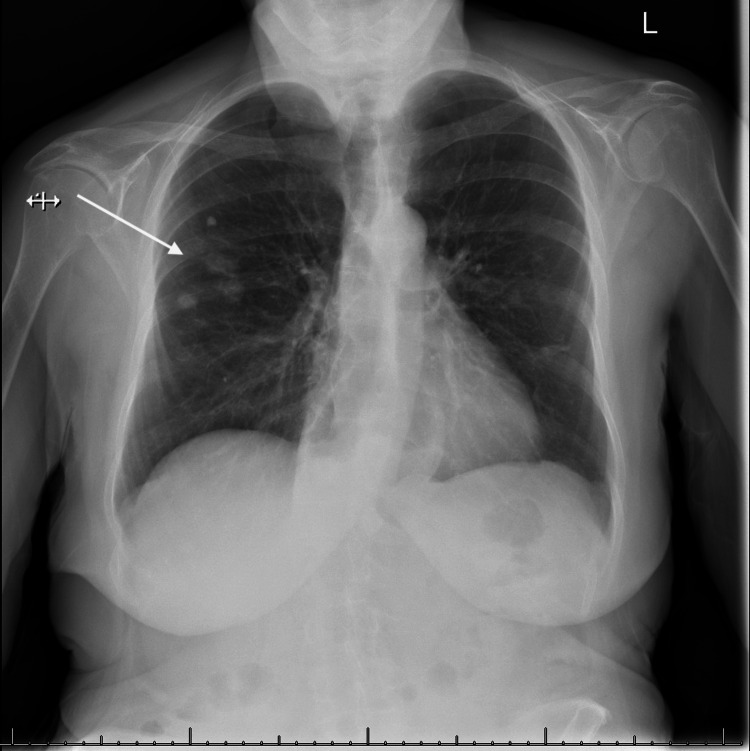
Chest x-ray showing nodular infiltrates in right lung fields

**Figure 2:**
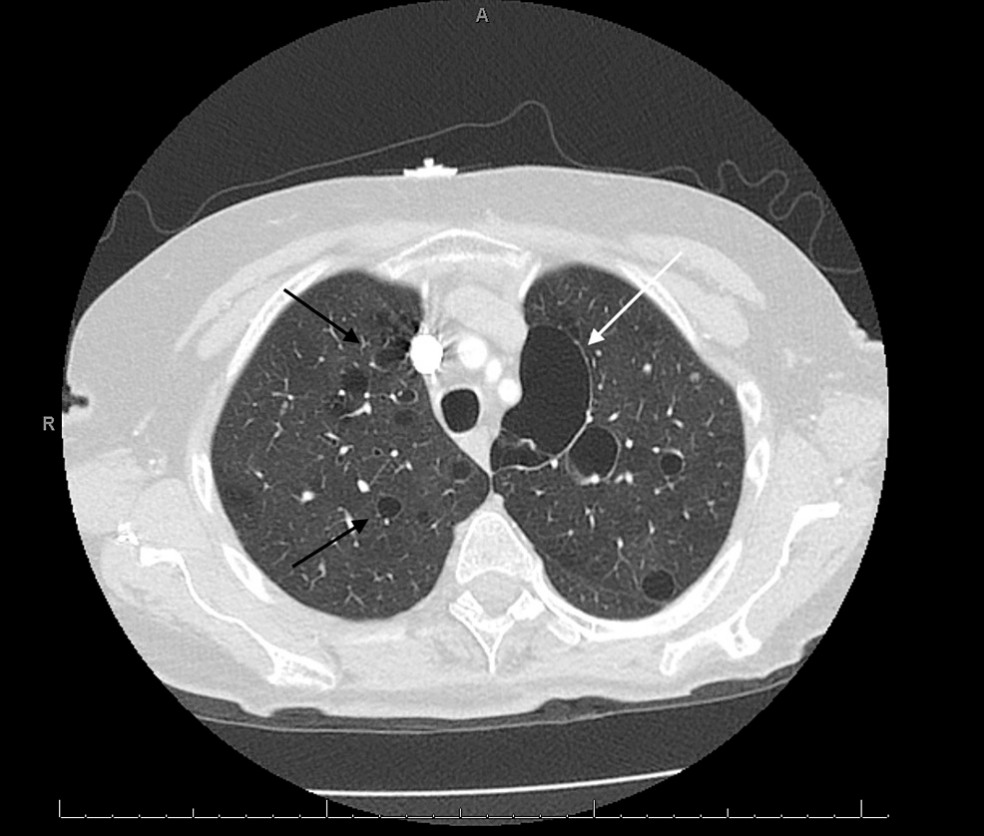
CT scan of the chest showing multiple nodular lesions in both lung fields (black arrows) and a large cystic mass in the left lung field (white arrow)

**Figure 3:**
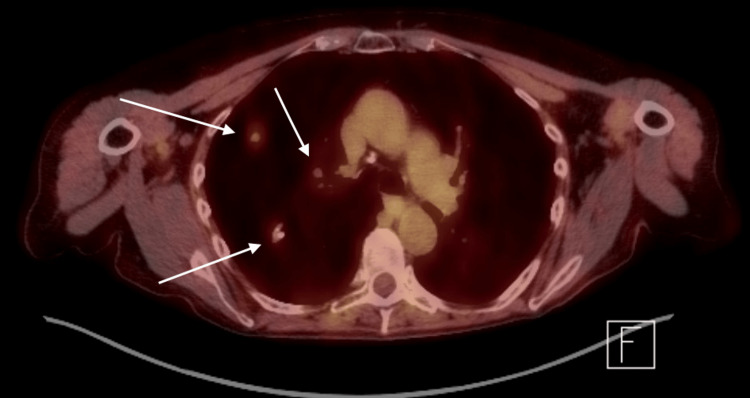
PET scan showing FDG avid lesions in lung fields (white arrows), raising a high suspicion for a neoplastic process PET: positron emission tomography; FDG: fluorodeoxyglucose

A 78-year-old lady presented to the emergency room with increasing shortness of breath and cough for the past two to three months. The patient had been using lubricant eye drops for the past many years for her dry eyes but did not have any past diagnosis of Sjogren syndrome. The patient had normal vital signs on presentation. Normal breath sounds were heard on auscultation. Complete blood count and basic metabolic profile were unremarkable. Chest x-ray showed multiple nodular lesions in the right lung fields (Figure 1). Computed Tomography (CT) scan of the chest showed numerous bilateral pulmonary nodules, including a spiculated 11 cm-sized mass in the left lower lobe, suspicious of malignancy (Figure 2). Positron emission tomography (PET)-CT scan showed fluorodeoxyglucose (FDG) activity in the lung nodules and FDG avid hilar region (Figure 3). Due to high suspicion of malignancy and for staging and characterization, the patient underwent surgical resection and biopsy of the right upper lobe. Pathologic specimen showed nodular amyloidosis, confirmed on Congo-red stain. Interestingly, there was no evidence of any neoplastic process in the resected specimen, despite high FDG activity on the PET scan. 

Due to the detection of amyloidosis, further workup was done due to high suspicion of autoimmune disorders. It showed positive antinuclear antibody (ANA) of 1:1280 with a speckled pattern, strongly positive Sjogren's syndrome A (SS-A)/Ro and Sjogren's syndrome B (SS-B)/La antibodies, and elevated ribonucleoprotein (RNP) antibodies. (Table 1). The monoclonal protein screen showed polyclonal paraproteinemia with elevated kappa and lambda light chains. There was also a slight isolated Immunoglobulin (Ig) G rise with normal levels of other Igs (Table 2).

**Table 1:** Autoimmune antibody screen test following the discovery of nodular amyloidosis on biopsy ANA: antinuclear antibody; CCP: cyclic citrullinated peptide; Ig: immunoglobulin; ds DNA: double-stranded DNA; RNP: ribonucleoprotein; SM: smooth muscle; SS: Sjogren syndrome

Laboratory	Value	Reference range and limits
ANA	1:1280, speckled	<1:80
CCP, IgG	<0.4	<0.4
anti ds DNA	negative	negative
C3 complement	135 mg/dL	90-230 mg/dL
C4 complement	24 mg/dL	10-51 mg/dL
RNP antibody	3.0 Elisa Units	<1.0 Elisa Units
SM antibody	0.2 Elisa Units	<1.0 Elisa Units
SS A/Ro antibody	>8.0 Elisa Units	<1.0 Elisa Units
SS B/La antibody	>8.0 Elisa Units	<1.0 Elisa Units
IgA, serum	316 mg/dL	70-400 mg/dL
IgM, serum	103 mg/dL	40-230 mg/dL
IgG, serum	1725 mg/dL	700-1600 mg/dL

**Table 2:** Monoclonal protein screen performed in the setting of amyloidosis Ig: immunoglobulin

Laboratory	Value	Reference range and limits
Free light chains ratio	2.19	0.26-1.65
Kappa light chains	69.1	3.3-19.4 mg/L
Lambda light chains	31.5	5.7-26.3 mg/L
IgG, serum	1,725	700-1,600 mg/dL
IgA, serum	316	70-400 mg/dL
IgM, serum	103	40-230 mg/dL
Protein, Total	7.4	6.58-8.51 g/dL
Albumin	3.70	3.73-5.65 g/dL
Alpha-1 globulins	0.34	0.13-0.45 g/dL
Alpha-2 globulins	0.90	0.37-0.93 g/dL
Beta globulins	0.81	0.69-1.29 g/dL
Gamma globulins	1.66	0.58-1.50 g/dL

In this scenario, the patient had persistent dry eyes and strongly positive SS-A and SS-B antibodies. Neoplastic processes were ruled out on biopsy, which leaves Sjogren syndrome causing and presenting as nodular pulmonary amyloidosis.

All the possible options including immunomodulators such as rituximab were discussed with the patient for the treatment of amyloidosis. After weighing the treatment options with the possible risk of immunosuppression, particularly in the ongoing coronavirus disease 2019 (COVID-19) pandemic, a decision was made not to start any treatment. The patient was followed up in the Rheumatology and Hematology clinic and she remained largely asymptomatic. She has not reported any other occurrence of symptoms since the initial presentation.

## Discussion

Lung amyloidosis has three main histologic presentations described as nodular pulmonary amyloidosis, tracheobronchial amyloidosis, and diffuse alveolar septal amyloidosis [6]. Nodular pulmonary amyloidosis usually represents localized AL or AL/heavy chain (AH) amyloidosis [7]. It is usually detected incidentally as the patients are largely asymptomatic on presentation. On detection, nodular amyloidosis is found to be isolated but may be present alongside a localized clonal proliferation of B-lymphocytes or plasma cells secreting light chains [8]. It has also been shown to be associated with an underlying lymphoproliferative disorder such as mucosa-associated lymphoid tissue (MALT) lymphoma [7]. 

Due to its frequent association with localized lymphoma and other lymphoproliferative disorders, surgical excision and biopsy are essential for the diagnosis of amyloidosis and exclusion of localized lymphoma, as management options vary. In the advent of worsening symptoms and extension of amyloidosis, surgical excision has been done successfully [9]. 

Sjogren syndrome is associated with multiple pulmonary disorders, most commonly being interstitial lung disease, bronchiolitis, chronic obstructive pulmonary disease, and reactive airway disease (Table 3) [10]. The presence of pulmonary nodular amyloidosis as a presentation of Sjogren syndrome is not widely reported. 

**Table 3:** Pulmonary complications of Sjogren syndrome DLCO: diffusing capacity of the lungs for carbon monoxide; FVC: forced vital capacity; HRCT: high-resolution computed tomography; ILD: interstitial lung disease; LIP: lymphocytic interstitial pneumonia; NSIP: non-specific interstitial pneumonia; OP: organizing pneumonia; PAH: pulmonary arterial hypertension; PFT: pulmonary function test; pSS: primary Sjogren’s syndrome; RHC: right heart catheterization; UIP: usual interstitial pneumonia.

Type of Lung Involvement	Main Clinical Features
Airways disorders	
Upper airways inflammation	Dryness in nasal and oropharyngeal mucosa finally leading to atrophy of the submucosal glands of the airway mucosa
Chronic obstructive pulmonary disease and reactive airway disease	Shortness of breath, chronic dry cough, or wheezing due to airway hyperresponsiveness. PFTs allow the distinction of the two patterns
Bronchiolitis (follicular bronchiolitis, chronic bronchiolitis, and bronchiolitis obliterans)	ITraditionally presents with cough and dyspnea. PFT findings may be normal or show either a restrictive or obstructive pattern. It appears as a reticular or reticulonodular pattern on HRCT
Bronchiectasis	Dry cough, isolated dyspnea, and hemoptysis in rare occasions. Patients with bronchiectasis have a higher frequency of respiratory infections and pneumonia
Parenchymal disease	
Interstitial lung disease	Non-productive cough and dyspnea are present in half of patients, the remaining are completely asymptomatic. On auscultation, it is characterized by fine bibasilar end-inspiratory “velcro-like” crackles. The most common ILD pattern is NSIP, followed by UIP, OP, and LIP. In early involvement, it is easy to observe a reduction of DLCO (alveolar inflammation) with a preserved FVC (normal lung volumes)
Lymphoma and pseudolymphoma	Cough, slowly progressive dyspnea, and traditional B symptoms (fever, night sweats, and weight loss) are characteristic of lung lymphoma. Parenchymal radiographic findings are often associated to mediastinal lymphadenopathy and pleural effusions Pseudolymphoma is generally asymptomatic, and it usually appears as a solitary nodule or mass
Pulmonary amyloidosis	Cough or dyspnea along with fatigue, weakness, hemoptysis, and pleuritic chest pain. Radiologically, it is characterized by large, calcified, randomly distributed, irregular, smooth-bordered nodules alone, or in association with LIP
Sarcoidosis	Asymptomatic or presenting with exertional dyspnea or dry cough. Heerfordt’s syndrome is defined by the characteristic parotid enlargement and the presence of uveitis. Radiographic abnormalities include bilateral hilar lymphadenopathy, pulmonary infiltrates, or fibrosis
Cystic lung disease	Mostly subclinical. Cysts are usually bilateral with the majority located in the middle lung
Pulmonary venous thromboembolism	Characterized by a lack of overt clinical symptoms in the early phase and by exertional dyspnea in more advanced disease. Physical examination could reveal a loud second pulmonary heart sound. PFTs commonly show normal lung volumes with a reduction in DLCO. Transthoracic Doppler echocardiography can be useful for screening for PAH however, RHC is required for definitive diagnosis and to orient between PAH different etiologies
Pulmonary arterial hypertension (PAH)	
Lymphocytic pleuritis	Rare manifestation in pSS characterized by chest, fever, cough, dyspnea, and exudative pleural effusion
Neuromuscular disease with secondary pulmonary involvement	Dyspnea, persistent episodes of chest pain, restrictive syndrome, and the absence of significant interstitial and/or pleural disease

In the current case, the presence of multiple nodular lesions in lung fields with FDG-avid lesions warranted the workup for malignancy with resection and biopsy. On the pathologic specimen, there was no evidence of neoplastic processes, and amyloidosis was confirmed with the Congo-red stain. With the autoimmune panel results and the presence of xerophthalmia, the association of nodular amyloidosis with Sjogren syndrome was established. 

This case report presents an uncommon association of Sjogren syndrome with nodular pulmonary amyloidosis. Further exploration of the pathophysiology of autoimmune disorders and associated inflammation can explain systemic amyloidosis but isolated organ amyloidosis in such conditions is uncommon. Further research and understanding of single-organ amyloid deposition in systemic conditions may provide a suitable explanation. 

## Conclusions

Isolated nodular pulmonary amyloidosis is a condition most commonly associated with lymphoproliferative malignancies. Along with its evaluation, clinicians also need to consider autoimmune disorders in the differential. Sjogren syndrome is one such condition that can infrequently present as nodular pulmonary amyloidosis. In that setting, management is generally conservative with symptomatic management.
